# Flawed conclusions on the Västerbotten Intervention Program by San Sebastian et .al

**DOI:** 10.1186/s12889-019-7444-3

**Published:** 2019-08-13

**Authors:** Yulia Blomstedt, Margareta Norberg, Nawi Ng, Lennarth Nyström, Kurt Boman, Göran Lönnberg, Hans Stenlund, Stig Wall, Lars Weinehall

**Affiliations:** 1 Department of Public Health and Clinical Medicine, Umeå University, SE-901 87 Umeå, Sweden; 2 Department of Epidemiology and Global Health, Umeå University, SE-901 87 Umeå, Sweden; 3 Research Unit Medicine-Geriatric Clinic, Skellefteå County Hospital, SE-931 86 Skellefteå, Sweden

**Keywords:** Prevention, Community intervention, Evaluation, CVD

## Abstract

An evaluation of Västerbotten Intervention Programme (VIP) was recently conducted by San Sebastian et al. (BMC Public Health 19:202, 2019). Evaluation of health care interventions of this kind require 1) an understanding of both the design and the nature of the intervention, 2) correct definition of the target population, and 3) careful choice of the appropriate evaluation method. In this correspondence, we review the approach used by San Sebastian et al. as relates to these three criteria. Within this framework, we suggest important explanations for why the conclusions drawn by these authors contradict a large body of research on the effectiveness of the VIP.

## Background

The effectiveness of the community-based prevention programmes implemented in a real-world setting is a subject of a continuous scientific and political debate. One of the reasons is the difficulty of evaluating such programmes [1].

The Västerbotten Intervention Programme (VIP) targets non-communicable diseases in the adult population of the Västerbotten county in northern Sweden. It is also one of the few programmes that has been shown by rigorous evaluation [2–13] to be consistently effective over 30 years of implementation. However, a recent publication by San Sebastian et al. [14] concludes that the intervention has not been effective. Herewith, we address the possible reasons for why San Sebastian et al.’s conclusions differ from the previous findings of other investigators.

1. San Sebastian et al. identify the intervention as having occurred in a single year (1994), which is incorrect

VIP was first piloted in the small municipality of Norsjö in 1985. The dissemination to all other primary care centres in Vasterbotten occurred gradually between 1985 and 1993, with the biggest municipalities joining early on and the vast majority of the primary health care centres actively working with the programme by 1991. This is explained in detail in a design article by Norberg et al. [15], which is also cited by San Sebastian et al. Therefore, it is not appropriate to treat 1987–1993 as the pre-intervention period as done by Sebastian et al. Nor is it appropriate to treat the years beyond 1994 as the post-intervention period, as it similarly implies that the VIP was implemented in a single year (see next section).

2) San Sebastian et al. include subjects who were not exposed to the VIP as being in the treatment group

While it is true that the entire 40–60 year old population of the county is theoretically eligible for VIP, the actual enrolment into the programme is phased in over time, with primary care centres only inviting those who turn exactly 40, 50 or 60 years old in each specific year. Therefore, 10 years elapsed before the entire eligible population was fully exposed to the intervention.

In their study, San Sebastian et al. [14] analysed the effect of the intervention on all individuals aged 40–74 between 1994 and 2013. However, this approach assesses treatment effect in many subjects who have never been eligible for the VIP. For example, an individual who was 74 in 1995 would have been between 40 and 60 years old between 1961 and 1981, which means he/she spent this entire period without being exposed to the intervention. Clearly, this is not a subject for whom treatment effect should be assessed.

Including individuals over 60 years of age when VIP started has other implications. In addition to being ineligible for the VIP, these individuals have a shorter life expectancy than those who were born later, which creates an obvious bias towards the null in the assessment of treatment effect. In fact, San Sebastian et al. acknowledge that inclusion of “non-eligible non-participants” in the analysis, who might have “worse-than-expected mortality trends”, “could completely offset the effect on mortality reduction”.

3) San Sebastian et al. use interrupted time series (ITS) as an analytical model

ITS is designed for evaluating interventions where 1) there is a clearly defined cut point corresponding to the time the intervention is implemented [16] and 2) the effects are expected to be felt relatively quickly after implementation, or after a clearly defined lag time [17]. Neither of these two conditions apply to the VIP.

First, both the gradual dissemination of the VIP between 1985 and 1993 and its time-lapsed recruitment strategy do not fit the scenario of a well-defined intervention cut point. Therefore, the arbitrary cut off year of 1994 chosen by San Sebastian et al. to represent the start of the intervention does not conform to the actual design of the study.

Second, it is well accepted throughout the public health community that interventions aimed at prevention do not exert their effects shortly after implementation. Rather, they evolve slowly over time. While VIP participation rates have been high [6] with only small social selection bias, it is not realistic to expect an immediate population effect of VIP on IHD and total mortality among the target population (participants + non-participants), the majority of whom are below 60 years of age.

### Other considerations

San Sebastian et al. find: “no evidence for a more positive development in Västerbotten following the implementation of the VIP” and conclude that: “the data do not support that the intervention has contributed to an additional reduction on IHD morbidity and mortality, above and beyond that which is already seen in neighbouring counties without similar programs”. To support their findings, the authors refer to publications similarly critical to community-based prevention programmes. However, grouping the VIP together with other prevention programs that use different strategies, structure and focus (such as The Scottish keep well health checks, NHS health checks and the Danish RCT Inter99) completely obscures these differences. Moreover, San Sebastian findings contradict both previous evaluations of the VIP [2–13], as well as evaluation of similar Swedish programmes [18] and evaluation of VIP by other independent groups [19].

In our previous evaluation of the VIP [12], the data were analysed according to an intention-to-treat strategy that identified a target group of participants as well as eligible non-participants. For the purpose of this correspondence, we took a closer look at the possible impact on the general population. A dramatic decrease in all-cause mortality could be observed both in Västerbotten and neighboring counties during the past 45 years, which, to a large extent, can be attributed to a reduction in cardiovascular diseases. Although the time trends are similar, it is evident that Västerbotten has performed better. While, for men age and time-standardized mortality in Västerbotten in the 70s was 4.5% higher and in the 80s 7.2% higher than the general Swedish male population (8.7 and 9.5% for women), it has been well below the national average since the mid-90s. Neither of the neighboring counties of Västernorrland or Norrbotten display this pattern (unpublished data).

### Limitation of our critique

The authors behind this correspondence do not constitute a neutral third party. Rather, we are the actual medical practitioners and researchers who have implemented or evaluated the VIP. Some of us, who have been with the program since its beginning, have devoted more than 30 years to this endeavour.

To ensure the quality of the programme, we have always welcomed new approaches to evaluating it and studying the underlying factors behind the effectiveness of prevention methods in general, particularly by independent groups. To facilitate this, we have made VIP data available through various collaborations, including the Ageing and Living Conditions Programme [20], SimSam [21] and the Northern Sweden Health and Disease Study [22] to name a few. In these cases however, we expect that researchers will devote sufficient time to understanding the programme design and the nature of the intervention. This is a key principle of the evaluation of health care interventions [23].

## Conclusions

As explained above, in the recently published evaluation of the VIP [14], the authors did not consider some of the complexities of the design of the VIP and it is on this basis that we question their methodology. This may be the reason that the conclusions of those authors are contradictory to a consensus of previous evaluations of the VIP.

## Data Availability

All data supporting the conclusions of this article are included within the article and list of references.

## Abbreviations

CVD: Cardio-vascular diseases
IHD: Ischemic heart disease
ITS: Interrupted time series
VIP: Västerbotten Intervention Programme
